# SUMO Modification of Stra13 Is Required for Repression of Cyclin D1 Expression and Cellular Growth Arrest

**DOI:** 10.1371/journal.pone.0043137

**Published:** 2012-08-14

**Authors:** Yaju Wang, Vinay Kumar Rao, Wai Kay Kok, Dijendra Nath Roy, Sumita Sethi, Belinda Mei Tze Ling, Martin Beng Huat Lee, Reshma Taneja

**Affiliations:** 1 Department of Physiology, National University of Singapore, Singapore, Singapore; 2 Division of Nephrology, National University Health System, Singapore, Singapore; Virginia Commonwealth University, United States of America

## Abstract

Stra13, a basic helix-loop-helix (bHLH) transcription factor is involved in myriad biological functions including cellular growth arrest, differentiation and senescence. However, the mechanisms by which its transcriptional activity and function are regulated remain unclear. In this study, we provide evidence that post-translational modification of Stra13 by Small Ubiquitin-like Modifier (SUMO) dramatically potentiates its ability to transcriptionally repress cyclin D1 and mediate G_1_ cell cycle arrest in fibroblast cells. Mutation of SUMO acceptor lysines 159 and 279 located in the C-terminal repression domain has no impact on nuclear localization; however, it abrogates association with the co-repressor histone deacetylase 1 (HDAC1), attenuates repression of cyclin D1, and prevents Stra13-mediated growth suppression. HDAC1, which promotes cellular proliferation and cell cycle progression, antagonizes Stra13 sumoylation-dependent growth arrest. Our results uncover an unidentified regulatory axis between Stra13 and HDAC1 in progression through the G_1_/S phase of the cell cycle, and provide new mechanistic insights into regulation of Stra13-mediated transcriptional repression by sumoylation.

## Introduction

Stra13, a member of the bHLH-O repressor subfamily is widely expressed both during embryonic development as well as in a number of adult tissues [1], [2]. In addition to being constitutively expressed in several cell types, its expression is up regulated in response to multiple stimuli including retinoic acid, TGFβ, serum deprivation, genotoxic agents and trichostatin A (TSA). Several gain of function and loss of function studies have shown its involvement in cellular differentiation programs, cell cycle progression, senescence, apoptosis, immune responses, tissue regeneration and circadian rhythms [3]–[17]. However, the molecular mechanisms through which Stra13 regulates these diverse biological responses are largely unclear. Previous studies have shown that Stra13 overexpression results in growth suppression, cell cycle arrest, and cellular senescence, which are important tumor suppression mechanisms [4], [6], [10], [12], [17]. Consistent with these observations, Stra13 expression is indeed down regulated in some tumors. Intriguingly however, it is also overexpressed in many cancers [2], [18]–[22]. Similarly, while Stra13 inhibits differentiation of some cell types, it promotes others [3], [8], [9]. The seemingly paradoxical functions of Stra13 could potentially occur by altered sub-cellular localization, or association with distinct co-factors in different cell types. Alternatively, post-translational modifications may allow it to rapidly and reversibly alter functions in diverse cellular contexts.

We and others have previously demonstrated that Stra13 associates with the co-repressor HDAC1 through its C-terminal repression domain that contains three α-helices [4] and regulates transcriptional repression of specific target genes [4], [23], [24]. However the mechanism by which HDAC1 regulates Stra13-dependent biological functions is unclear.

SUMO (Small Ubiquitin-related Modifier) modification, or sumoylation, is an important post-translational modification that modulates the biological functions of proteins [25], [26]. Sumoylation is a highly dynamic process, whereby SUMO is covalently conjugated to an obligatory lysine in canonical ψKXE SUMO motifs (where ψ is a hydrophobic amino acid, K is the acceptor lysine for covalent attachment of SUMO, and X is any residue, and E is glutamic acid) in the substrate. Sumoylation is a three-step reaction consisting of SUMO activation, transfer, and ligation that are catalyzed by E1 heterodimeric enzyme (SAE1/SAE1), E2 enzyme (Ubc9) and E3 SUMO ligases, of which the Protein Inhibitor of Activated Stats (PIAS) proteins have been well-characterized [27], [28]. Protein sumoylation is readily reversed by cellular isopeptidases or Sentrin/SUMO-specific proteases (SENP, SUSP), which cleave SUMO from its substrate. Unlike ubiquitination, which usually facilitates protein degradation, sumoylation results in pleiotropic functional consequences that include changes in subcellular localization, protein stability, alterations in DNA-binding and transcriptional activity. Transcription factors, co-activators and co-repressors are predominant targets of sumoylation, which alters their activity resulting in changes in gene expression and function [29], [30].

In this study, we demonstrate that Stra13 can be SUMO modified at conserved residues Lys 159 and Lys 279 that is enhanced by the SUMO E3 ligases PIAS3 and PIAS1. Mutation of these target residues, or co-expression of the SUMO protease SENP1 with wild type Stra13, impairs its ability to repress cyclin D1 expression and attenuates its function as a growth suppressor. In addition, mutation of sumoylation sites reduces association of Stra13 with HDAC1, which plays an essential role in cell cycle progression. HDAC1 inhibits Stra13 sumoylation in a deacetylase-activity dependent manner and blocks its anti-proliferative effects. Together these studies identify sumoylation as a key post-translational modification that modulates Stra13 transcriptional repression activity and function in cell cycle arrest.

## Results

### Stra13 Sumoylation is Enhanced by PIAS3 and PIAS1

**Figure 1:**
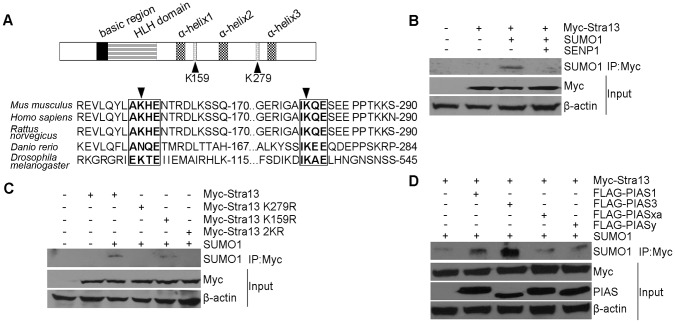
Stra13 is sumoylated. (A) Schematic representation of the Stra13 domain structure (upper panel). The basic and HLH domains are shown along with three α-helices in the C-terminal repression domain. Potential sumoylation acceptor lysines at 159 and 279 (K159 and K279) are indicated. Numbers indicate amino acid residues in the mouse Stra13 cDNA. Alignment of Stra13 cDNA from various species revealed a highly conserved SUMO consensus motif IKQE, and a somewhat less conserved motif AKHE that are highlighted. K159 and K279 are indicated by arrowheads (lower panel). (B) Cells were co-transfected with Myc-Stra13, SUMO1 and SENP1 as indicated. Lysates were immunoprecipitated with Myc-agarose beads followed by immunoblotting with anti-SUMO1 antibody. Input shows expression of Stra13 using anti-Myc antibody. β-actin served as a loading control. (C) Cells were co-transfected with Myc-Stra13, or point mutants (Stra13 K279R, Stra13 K159R, Stra13 2KR) together with SUMO1. Cell lysates were immunoprecipitated with Myc-agarose beads and the immunoprecipitates were subjected to western blotting with anti-SUMO1 antibody. (D) Myc-Stra13 and SUMO1 were expressed along with Flag-PIAS1, PIAS3, PIASxα, or PIASy as indicated. Lysates were immunoprecipitated with Myc- agarose beads followed by western blotting with anti-SUMO1 antibody. Lysates (input) were probed for Stra13 and PIAS.

We have previously demonstrated that the co-repressor HDAC1 interacts with the C-terminal region of Stra13 spanning amino acid residues 111–343 (Fig. 1A; and 4). Alignment of this region from several species revealed two potential sumoylation motifs AKHE and IKQE. K279 within the IKQE motif was phylogenetically conserved, whereas K159 within AKHE was less conserved through various species (Fig. 1A). To examine whether Stra13 undergoes sumoylation, we transfected cells with constructs encoding Myc-Stra13 in the absence or presence of SUMO1. Lysates were immunoprecipitated with Myc-agarose beads followed by western blotting with anti-SUMO1 antibody. A putative sumoylated band was detected in the presence of SUMO1 (Fig. 1B). To examine whether the slower migrating band corresponds to sumoylated Stra13, we co-transfected the Sentrin-specific protease (SENP), which is able to remove SUMO conjugates from substrates. In the presence of SENP1, sumoylation was abrogated, confirming that Stra13 is indeed sumoylated in cells. To examine whether K159 and K279 serve as acceptor sites for sumoylation, we generated lysine (K) to arginine (R) point mutants at each site individually (Stra13 K159R and Stra13 K279R respectively) and together (2KR) by site-directed mutagenesis. Cells were transfected with Stra13, Stra13 K159R, Stra13 K279R as well as the double mutant Stra13 2KR (Fig. 1C). Immunoprecipitation and western blotting analysis revealed that in presence of SUMO1, both Stra13 and Stra13 K159R mutant were sumoylated. In contrast, neither Stra13 K279R, nor Stra13 2KR were sumoylated, suggesting SUMO conjugation occurs predominantly at K279. The PIAS protein family act as E3 SUMO ligases and enhance sumoylation of target proteins [28]. To examine whether PIAS proteins modulate Stra13 sumoylation, we co-transfected cells with Myc-Stra13, SUMO1 and Flag-PIAS1, PIAS3, PIASxα, and PIASy. Cell lysates were immunoprecipitated with Myc-agarose beads, and analyzed by western blotting with anti-SUMO1 antibody. Both PIAS3 and PIAS1 enhanced Stra13 sumoylation, whereas expression of PIASxα and PIASy had a minimal impact (Fig. 1D).

### Stra13 Sumoylation is Required for its Anti-proliferative Effects

**Figure 2:**
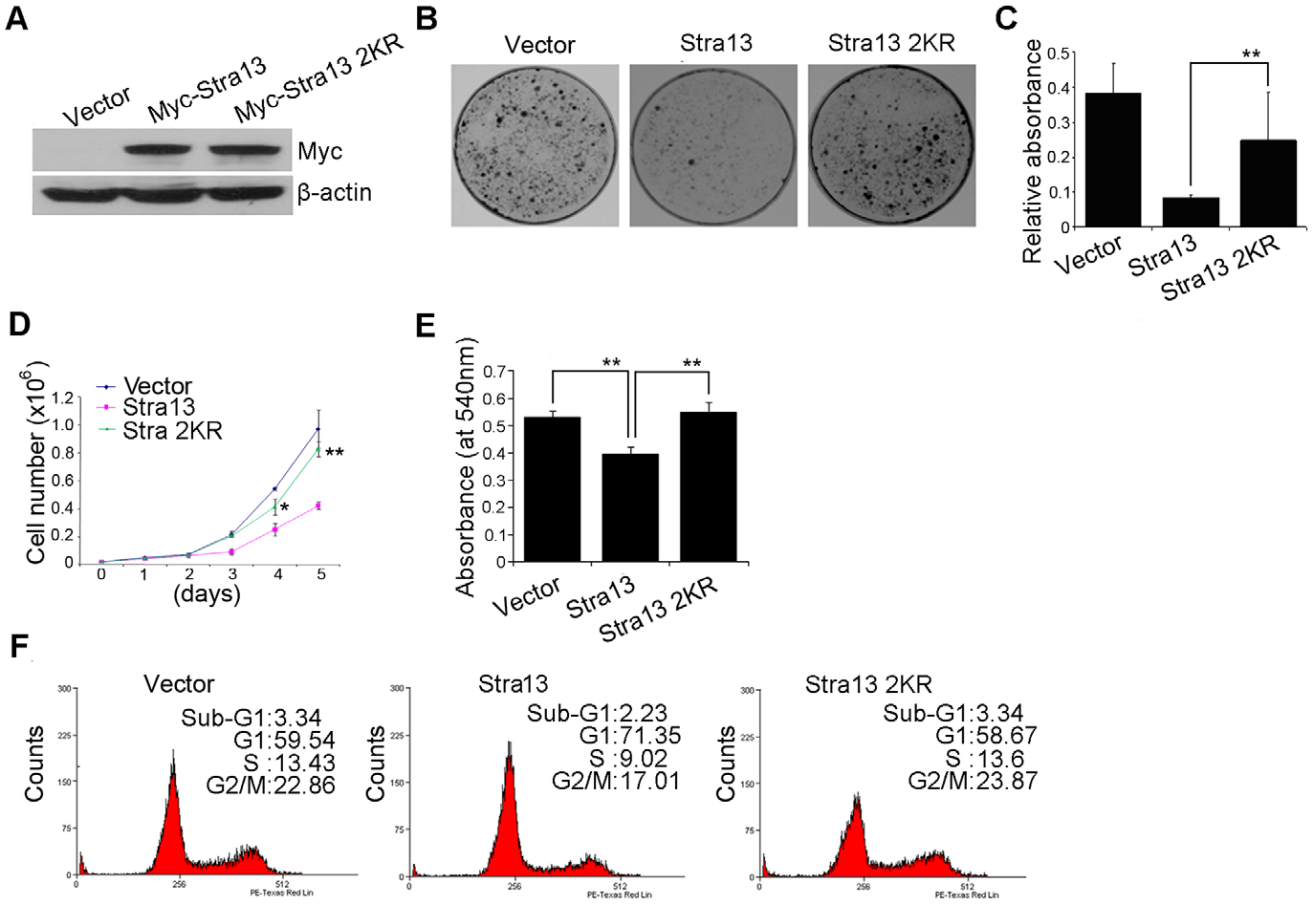
Mutation of sumoylation sites abrogates Stra13-mediated growth suppression. (A) NIH3T3 cells were co-transfected with Stra13 or Stra13 2KR together with a puromycin resistance plasmid. Empty vector (pCS2) was transfected in control cells (Vector). Stra13 expression was determined by western blotting using anti-Myc antibody. (B–C) Colony forming assays were performed with control, Stra13 and Stra13 2KR cells. Colonies were stained with crystal violet 14 days later. Data are representative of three independent experiments (B). Crystal violet dye was extracted and the absorbance measured at a wavelength of 570 nm. The error bars indicate standard deviations for triplicate wells in each experiment (C). (D) Growth of NIH3T3 cells expressing vector alone, Stra13 and Stra13 2KR was evaluated over a five-day period. Cell numbers at each time are represented as mean ±SD. (E) Stra13^−/−^ MEFs were transfected at passage 5 with equivalent amounts of Stra13 and Stra13 2KR. Cell viability was measured three days later by MTT assays. (F) Cell cycle profile of control (Vector), Stra13 and Stra132KR cells was determined by PI staining and FACS analysis. Representative histograms of cell cycle profiles in cells expressing vector alone, Stra13 and Stra13 2KR. The result shown is representative of three independent experiments.

Stra13 mediates growth suppression in a number of cell types and has also been implicated in cellular senescence [4], [6], [10], [12]. We therefore examined whether sumoylation impacts Stra13-mediated growth arrest. NIH3T3 cells were co-transfected with Stra13, or Stra13 2KR, along with pD503, which confers resistance to puromycin. Western blot analysis showed equivalent expression of Stra13 and Stra13 2KR (Fig. 2A). After selection, cells were seeded at a low density and analyzed for colony formation two weeks later. Consistent with our previous studies [4], overexpression of Stra13 resulted in significant reduction in colony numbers compared with vector-transfected cells (Fig. 2B, C). Interestingly, in contrast to wild type Stra13, Stra13 2KR was unable to inhibit colony formation (Fig. 2B, C). To examine the underlying mechanisms, we measured proliferation of cells expressing Stra13 and Stra13 2KR relative to vector controls. Stra13 overexpressing cells resulted in reduced cell numbers over a five-day period, whereas Stra13 2KR expressing cells proliferated similar to control cells (Fig. 2D). Consistently, re-expression of wild type Stra13 but not Stra13 2KR in mouse embryonic fibroblast (MEFs) derived from Stra13^−/−^ mice [5] led to growth suppression (Fig. 2E). We then examined the cell cycle profile of Stra13 and Stra13 2KR overexpressing cells by flow cytometry (Fig. 2F). Compared to controls, Stra13 expressing cells exhibited delayed progression in the G_1_/S phase of the cell cycle, resulting in G_1_ arrest. In contrast, the cell cycle profile of Stra13 2KR cells was similar to controls. However, expression of either protein had no impact on the sub-G_1_ phase. Together these results suggest that reduced colony formation upon Stra13 overexpression is due to reduced proliferation rates and G_1_ arrest rather than increased apoptosis.

**Figure 3:**
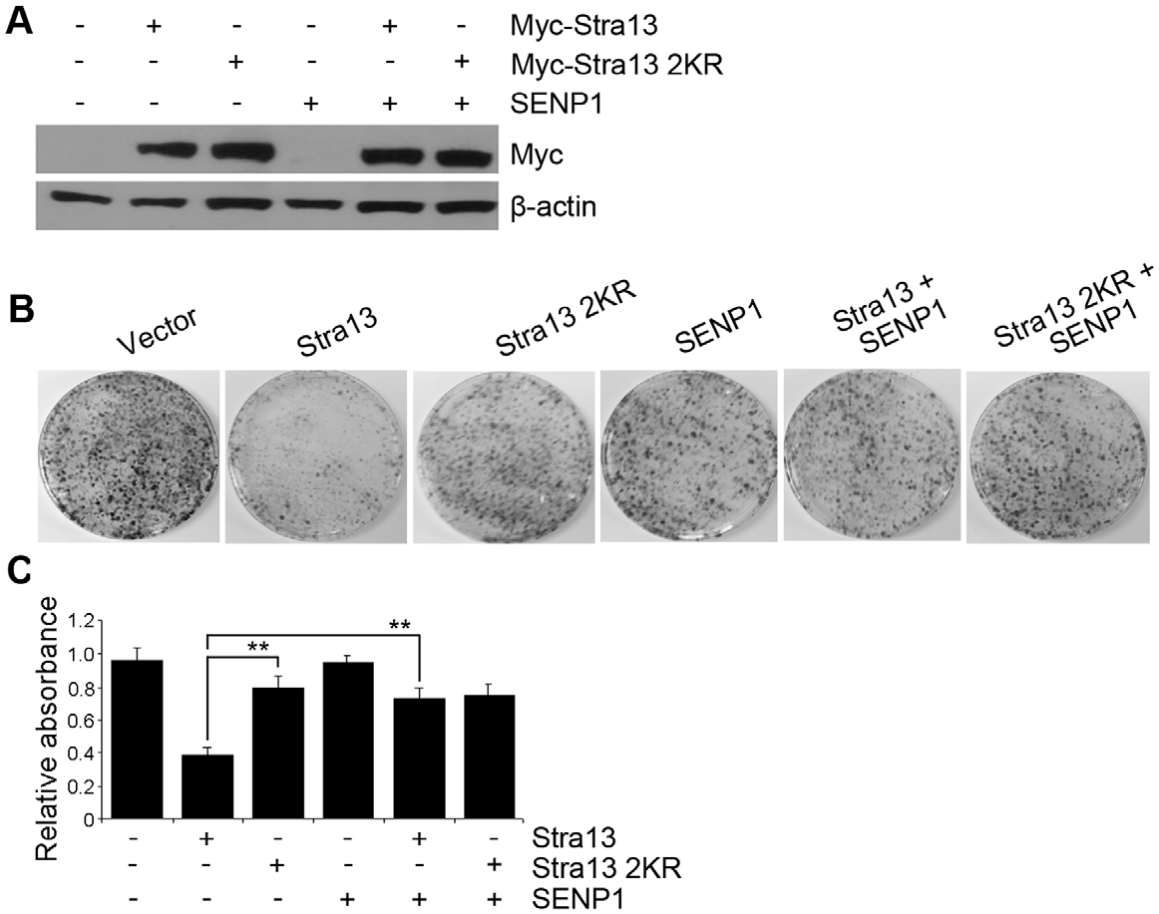
Sumoylation is essential for Stra13-dependent growth inhibition. (A) Lysates of NIH3T3 cells transfected with Myc-Stra13, Stra13 2KR and SENP1 were immunoblotted with anti-Myc antibody. (B–C) After selection, colony assays were performed and colonies were stained with crystal violet. Representative plates are shown (B). The mean relative absorbance after extraction of crystal violet stain from plates in shown in C. Error bars indicate mean ±SD.

The inability of Stra13 2KR mutant to mediate growth suppression suggested that sumoylation may be involved in the anti-proliferative effects of Stra13. To determine the impact of sumoylation on Stra13-mediated growth inhibition, we asked whether inhibition of Stra13 sumoylation recapitulates the phenotype of Stra13 2KR expressing cells. To examine this possibility, we performed colony assays in cells expressing the SUMO protease SENP1 along with equivalent levels of Stra13 and Stra13 2KR (Fig. 3A). Co-expression of SENP1 reversed the anti-proliferative effect of Stra13 whereas, as expected, Stra13 2KR was insensitive to SENP1 (Fig. 3B–C) confirming that sumoylation of Stra13 is indeed critical in mediating growth arrest in fibroblast cells.

### SUMO Modification of Stra13 does not Affect its Subcellular Localization but Enhances its Ability to Repress Cyclin D1

**Figure 4:**
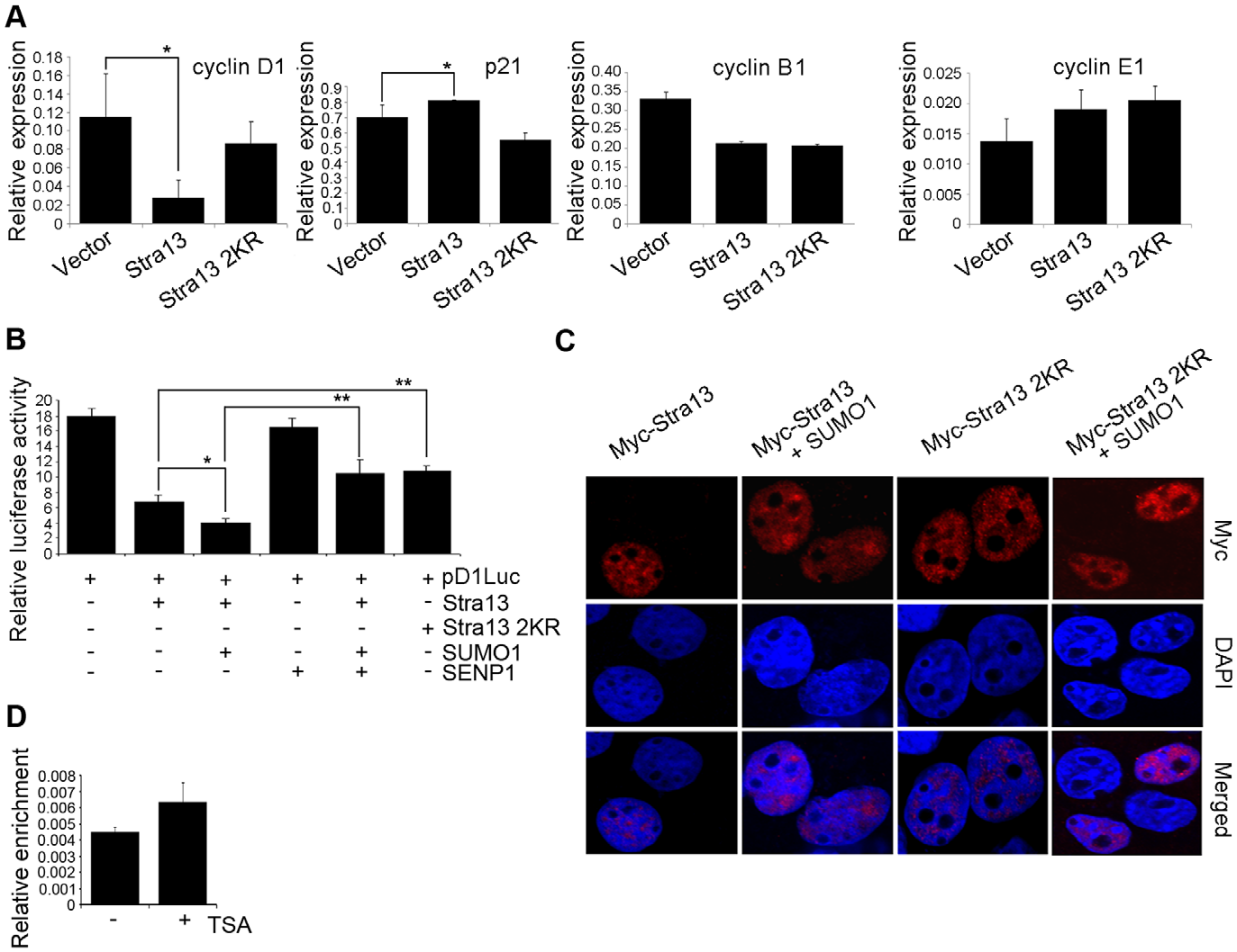
Sumoylation regulates Stra13 transcriptional activity but not its subcellular localization. (A) mRNA levels of cyclin D1, p21^Cip/WAF^, cyclin B1, and cyclin E1 were analyzed by Q-PCR in vector, Stra13 and Stra13 2KR cells. (B) Cells were transfected with the cyclin D1 promoter reporter pD1luc (100 ng) together with Stra13 (25 ng), Stra13 2KR (25 ng), SUMO1 (25 ng) or SENP1 (25 ng), as indicated. Cells were harvested 48 hr after transfection, and assayed for luciferase activity. (C) COS-7 cells were transfected with Stra13 and Stra13 2KR alone or together with SUMO1. Cells were stained with anti-Myc antibody. Nuclei were stained with DAPI. Error bars indicate mean ±SD. (D) NIH3T3 cells were left untreated or treated with TSA. ChIP assays were done to determine Stra13 occupancy on the cyclin D1 promoter.

To examine the molecular basis underlying Stra13-mediated G_1_ arrest, the expression of endogenous cyclin D1 and p21 that regulate G_1_/S transition was analyzed by Q-PCR. Stra13 significantly inhibited cyclin D1 expression, and up-regulated the levels of p21^Cip/WAF^ (Fig. 4A). In contrast, Stra13 2KR expressing cells did not show a significant change in the expression of either gene relative to control cells. Cyclin B1 and cyclin E1 expression was similarly regulated in cells expressing Stra13 and Stra13 2KR. Since sumoylation typically enhances transcriptional repression, we examined whether it is required for Stra13-mediated repression of cyclin D1 expression. A cyclin D1 promoter reporter [31] was co-expressed with Stra13, SUMO1 and SENP1. Consistent with repression of endogenous cyclin D1 expression, Stra13 repressed the cyclin D1 reporter, which was further augmented in presence of SUMO1, and attenuated in the presence of SENP1 (Fig. 4B). Stra13 2KR was unable to repress cyclin D1 promoter to the levels achieved with Stra13, confirming that sumoylation is relevant in Stra13-mediated repression of cyclin D1 expression. Since sumoylation can regulate the subcellular localization of target proteins [25]–[27], we examined whether the inability of Stra13 2KR to repress cyclin D1 was due to altered cellular localization. Stra13 and Stra13 2KR transfected cells were immunostained with anti-Myc antibody, and visualized by confocal microscopy. Both proteins showed nearly identical patterns of nuclear localization that was independent of sumoylation sites (Fig. 4C). Similarly, no differences were apparent between wild type and Stra13 2KR in the presence of SUMO1. Thus, the inability of Stra13 2KR to transcriptionally repress cyclin D1 is not due to altered subcellular localization. To determine whether cyclin D1 is directly regulated by Stra13, we performed chromatin immunoprecipitation (ChIP) assays in NIH3T3 cells that were left untreated or treated with trichostatin A (TSA) which causes growth arrest [4]. Binding of endogenous Stra13 was evident on the cyclin D1 promoter both in the absence and presence of TSA treatment (Fig. 4D). These findings are consistent with a recent study [17] demonstrating that cyclin D1 is a Stra13 target gene.

### HDAC1 Regulates Stra13 Sumoylation, Cyclin D1 Repression and Growth Arrest

**Figure 5:**
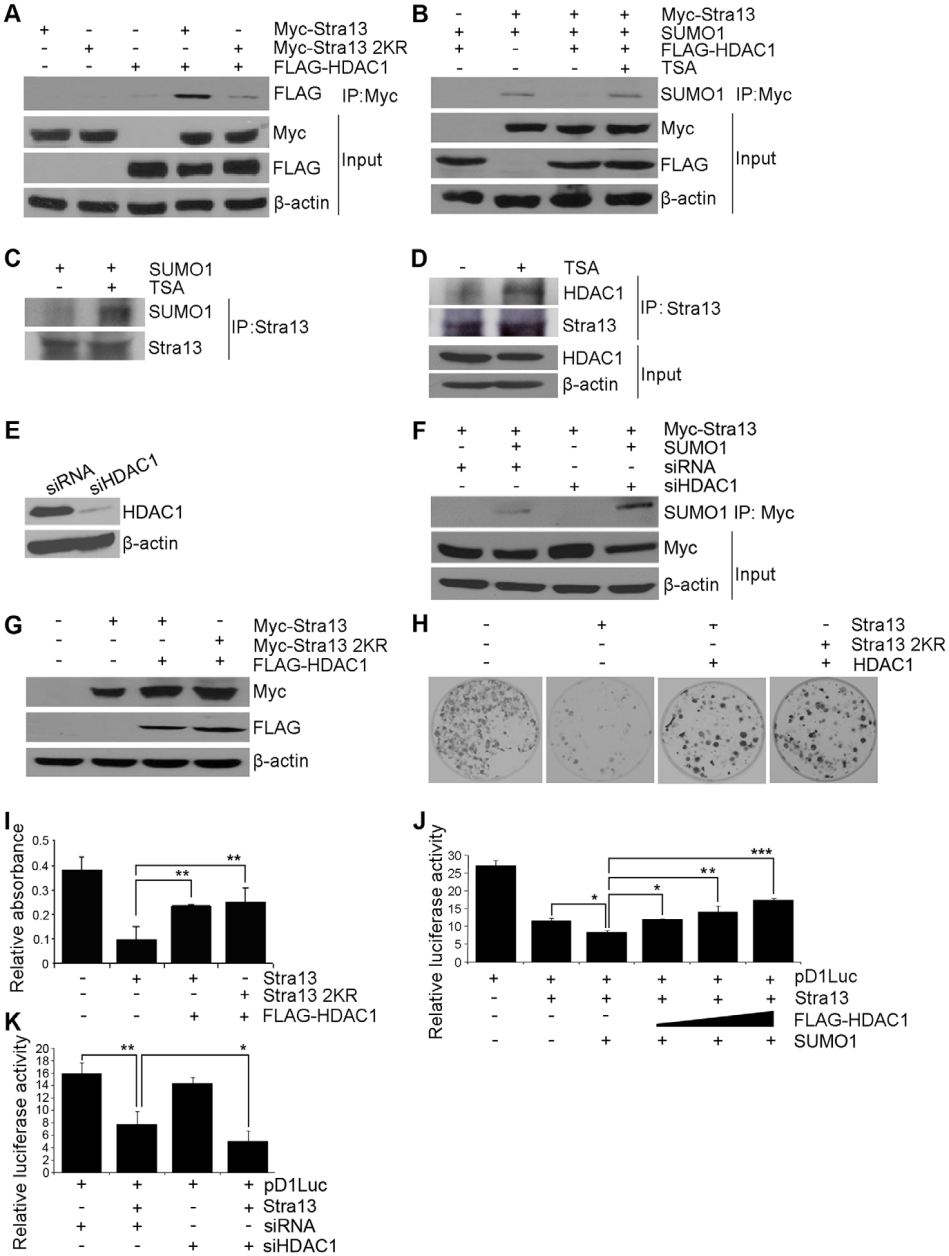
HDAC1 regulates Stra13 sumoylation. (A) Cells were co-transfected with plasmids expressing Flag-HDAC1 and Myc-Stra13 or Stra13 2KR. 48 hr after transfection, lysates were immunoprecipitated with Myc-agarose beads and analyzed for interaction by western blotting with anti-Flag antibody. (B) Cells were co-transfected with constructs encoding Myc-Stra13, Flag-HDAC1 and SUMO1. TSA was added at a concentration of 300 nM. Cell lysates were immunoprecipitated with Myc-agarose beads followed by western blotting with anti-SUMO1 antibody. (C–D) NIH3T3 cells were left untreated (−) or treated (+) with TSA. Endogenous Stra13 was immunoprecipitated and analyzed for sumoylation (C), as well as for association with HDAC1 (D). (E–F) Down-regulation of endogenous HDAC1 expression in siHDAC1 cells compared to control cells was examined by western blotting (E). Control and siHDAC1 cells were transfected with Stra13 and SUMO1. Lysates were immunoprecipitated as indicated and analyzed with anti-SUMO1 antibody (F). (G–I) Cells were co-transfected with Flag-HDAC1 and Myc-Stra13 or Stra13 2KR. Lysates were subject to western blotting with anti-Myc and anti-Flag antibodies to detect expression of Stra13 and HDAC1 (G). Colony assays were performed, and representative plates stained with crystal violet are shown (H). Colony assays were quantified by measuring the absorbance of extracted crystal violet dye at 570 nm (I). (J) Cells were transfected with the pD1luc reporter (100 ng) with Myc-Stra13 (25 ng) and SUMO1 (25 ng) in the presence of increasing amounts of HDAC1 (25, 50 and 100 ng). 48 hr later, luciferase activity was assayed. (K) siHDAC1 cells and controls were transfected with pD1luc in the absence and presence of Stra13. Luciferase activity was measured 48 hr later. Error bars indicate mean ±SD.

As Stra13 sumoylation sites are located within the HDAC1 interaction region, we tested whether the association between HDAC1 and Stra13 is SUMO-dependent. Cells were transfected with Stra13 or Stra13 2KR mutant together with Flag-HDAC1. Lysates were immunoprecipitated with Myc-agarose beads followed by western blotting with anti-Flag antibody (Fig. 5A). As previously reported, Stra13 interacted with HDAC1 [4], and consistent with a recent report [32], sumoylation-defective Stra13 2KR interacted less efficiently with HDAC1 compared to wild type Stra13. Several studies have demonstrated that HDACs modulate sumoylation of proteins. To investigate whether HDAC1 modulates Stra13 sumoylation, we co-expressed the two proteins. Interestingly, Stra13 sumoylation was almost completely abolished by HDAC1. Moreover, TSA (a histone deacetylase inhibitor) reversed HDAC1-mediated inhibition of Stra13 sumoylation (Fig. 5B). To validate these findings, endogenous Stra13 was immunoprecipitated from cells in the absence and presence of TSA treatment. Indeed, Stra13 sumoylation (Fig. 5C), and its association with HDAC1 (Fig. 5D), was enhanced in TSA treated cells. To further examine the role of endogenous HDAC1 in Stra13 sumoylation, its expression was down-regulated with HDAC1 specific siRNA (siHDAC1). Control cells were transfected with scrambled siRNA (siRNA). The down-regulation of HDAC1 expression in siHDAC1 cells (Fig. 5E) led to enhanced Stra13 sumoylation compared to controls (Fig. 5F) demonstrating that endogenous HDAC1 regulates Stra13 sumoylation.

In contrast to the anti-proliferative effect of Stra13, HDAC1 is known to promote proliferation via regulation of G_1_/S progression. Given its impact on sumoylation, we examined whether HDAC1 antagonizes Stra13-dependent growth suppression that is sumoylation-dependent. NIH3T3 cells were co-transfected with equivalent amounts of Stra13 and Stra13 2KR along with HDAC1 (Fig. 5G). In its presence, Stra13-mediated growth suppression was abrogated and phenotypically resembled Stra13 2KR cells (Fig. 5H, I). To further investigate whether the loss of growth suppression occurs due to an impact on cyclin D1, we performed reporter assays. In presence of HDAC1, repression of the cyclin D1 promoter by Stra13 and SUMO1 was attenuated in a dose-dependent manner (Fig. 5J). Conversely, in siHDAC1 cells, Stra13-mediated repression of cyclin D1 was augmented compared to controls (Fig. 5K) Together, these results demonstrate that HDAC1 inhibits Stra13 sumoylation, and consequently its ability to repress cyclin D1 that is essential for growth suppression in fibroblast cells.

## Discussion

In this study we have identified sumoylation as a key modification that impacts Stra13-mediated transcriptional repression with an overt impact on its function in cell cycle arrest. Mutation of sumoylation sites attenuates the anti-proliferative effect of Stra13, at least in part by abrogating its ability to inhibit cyclin D1 expression.

Post-translational modifications play a significant role in the regulation of transcription factors. Co-regulator proteins can either promote or inhibit these modifications. SUMO modification of many transcription factors appears to correlate with transcriptional repression, which may reflect altered protein-protein interactions. For instance, association with co-repressors such as HDACs is generally enhanced by sumoylation, and conversely interaction with co-activators is reduced [30], [33]. Consistent with altered recruitment of co-regulators, sumoylated Stra13 efficiently interacted with HDAC1, whereas the sumoylation defective mutant Stra13 2KR exhibited reduced association. HDACs enhance sumoylation of target proteins such as MEF2 and HIC1 that may occur via deacetylation of lysine residues, allowing them to be subsequently modified by sumoylation [34], [35]. Intriguingly however, HDAC1 inhibits Stra13 sumoylation and its ability to repress cyclin D1 thereby countering its anti-proliferative impact in fibroblast cells. HDAC1-mediated inhibition of Stra13 sumoylation and cyclin D1 repression is consistent with the opposing functions of the two proteins in cellular proliferation. Stra13 has been reported to repress cyclin D1 levels that correlate with its ability to mediate G_1_ arrest and cause growth suppression. In contrast, HDAC1 and HDAC2 promote cellular proliferation and cell cycle progression by inhibiting the cyclin dependent kinases (CDK) p21^WAF1/CIP1^ and p57^Kip2^ through direct regulation of their promoters resulting in transcriptional repression [36], [37]. Correspondingly, mouse embryonic fibroblasts lacking HDAC1 and HDAC2 are arrested in G_1_, and express elevated levels p21^WAF1/CIP1^ and p57^Kip2^. Moreover, increased expression of HDACs has been found in several cancers confirming their roles in cellular proliferation [38], [39]. Our studies suggest that in addition to direct regulation of CDK levels, HDAC1 may indirectly enhance proliferation by blocking growth suppressive signals via desumoylation of Stra13 relieving repression of cyclin D1. Given the impact of HDAC1 activity on Stra13 sumoylation, it is conceivable that in cells that overexpress both proteins, Stra13 may exist in a desumoylated state, and unable to block cellular proliferation allowing cells to bypass its growth suppressive function. The mechanism by which HDAC1 inhibits Stra13 sumoylation remains to be investigated. Since TSA can antagonize the effect of HDAC1 on Stra13 sumoylation, endogenous deacetylase activity is involved and may reflect a requirement for acetylation-dependent sumoylation similar to PML [40]. Alternatively, histone deacetylation by HDAC1 may release promoter bound sumoylated Stra13, which could then become accessible for desumoylation. Nonetheless, our studies demonstrate sumoylation is an important mechanism by which Stra13 transcriptional activity and function is modulated. Such post-translational modifications may underlie the seemingly paradoxical functions of Stra13 to either promote or inhibit cellular differentiation and growth in different contexts.

## Materials and Methods

### Plasmids, Mutagenesis, and HDAC1 siRNA

Flag-mPIAS1, Flag-mPIAS3, Flag-mPIASxα, Flag-mPIASy, SUMO1, SENP1, Flag-HDAC1, and pD1luc harboring the cyclin D1 promoter have been described [4], [31], [41]. pCS2-Myc-Stra13 was derived from pCS2-Flag-Stra13 by PCR using primers 5′-GAA TTC ATG GAG CAG AAA CTC ATC TCT GAA GAG GAT CTG GAA CGG ATC CCC AGC GCG-3′ and 5′-GAA TTC TTA GTC TTT GGT TTC TAA GTT-3′. The PCR product was TA-cloned into pCRII (Invitrogen), and then subcloned into the EcoR1 site of pCS2. K279R, K159R, and 2KR mutants were generated from pCS2-Myc-Stra13 using the QuickChange™ site-directed mutagenesis kit (Stratagene). Primers used for generating Stra13K279R are: 5′-GTC AGC ACA ATT AGG CAA GAA TCC GAA-3′ and 5′-TTC GGA TTC TTG CCT AAT TGT GCT GAC-3′; and for generating Stra13 K159R are: 5′-CAG TAC CTG GCG AGG CAT GAG AAC ACT-3′ and 5′-AGT GTT CTC ATG CCT CGC CAG GTA CTG-3′. The entire cDNA was sequenced to confirm the presence of directed mutations. For HDAC1 knockdown, NIH3T3 cells were transfected with 100 nM siRNA specific for mouse HDAC1 (Ambion); or with control scrambled siRNA using Lipofectamine RNAiMAX (Invitrogen). The efficiency of knockdown was determined using anti-HDAC1 antibody.

### Cell Culture and Transient Transfections

HEK293 cells, COS-7 cells NIH3T3 and cells were cultured in Dulbecco’s modified Eagle’s medium (DMEM) supplemented with 10% fetal bovine serum (HEK293), 10% calf serum (COS-7) and 10% bovine serum (NIH3T3) respectively. MEFs were cultured as described [5]. Transfections were performed using Lipofectamine Plus reagent according to the manufacturer’s instructions (Invitrogen).

### Immunoprecipitation and Western Blot Analysis

Cells were washed twice in cold PBS, lysed in 50 mM Tris-HCl pH 8.0, 50 mM NaC1, 1 mM EDTA, 0.1% Triton X-100, 0.5 mM PMSF and protease inhibitors (Roche). To detect sumoylation, 20 mM N-ethylmaleimide (Sigma) was added to lysis buffer. Protein concentrations were determined by the Bradford method (BioRad). Equal amounts of total protein were loaded for western blotting. Lysates were incubated with Myc-agarose beads (Sigma) in pull down buffer (as described above), and precipitates analyzed by western blotting using the following antibodies: anti-SUMO1 (1∶200 Zymed), anti-Flag (1∶5000 Sigma), anti-Myc (1∶2000 Roche) and anti-β-actin (1∶10,000 Sigma). For endogenous IP, 1.5 mg lysate was immunoprecipitated with 2 µg anti-Stra13 antibody (Bethyl Laboratories and Novus Laboratories), and immunoblotted with anti-SUMO1 antibody and anti-HDAC1 antibody respectively (Upstate).

### Proliferation, Cell Cycle Analysis, and Colony Suppression Assays

NIH3T3 cells or MEFs were transfected with Stra13 or Stra13 2KR with pD503 that confers resistance to puromycin. Control cells were transfected with empty vector and pD503. 24 hours (hr) after transfection, cells were selected with 1.2 µg/ml puromycin for three days. Selected cells were used for the following assays:

#### Proliferation/Viability assays

Cells were seeded at 1×10^4^ per well in 6-well plates in triplicates. Attached cells were trypsinized and proliferation was measured by counting cells daily over a period of five days. Alternatively, 3000 cells were seeded in 96-well plates and 72 hr later, MTT assays were performed using MTT cell proliferation assay kit (Invitrogen).

#### Cell cycle

Selected cells were seeded at a density of 5×10^5^/10-cm plates. 24 hr later, cells were fixed with cold 70% ethanol. Twenty thousand cells were acquired and analyzed for DNA content by propidium iodide staining (50 µg/ml) as described previously [4], [42]. Cell cycle distribution was analyzed by flow cytometer (Becton Dickinson) using WINMDI software.

#### Colony suppression assays

Colony assays were done as described [4], [42]. Briefly cells were seeded at 1×10^3^/10-cm plate. Two weeks later, colonies were fixed with 70% ethanol stained with 0.02% crystal violet and photographed. For quantification, the dye was extracted in 1% SDS and the absorbance read at 570 nm.

#### Chromatin immunoprecipitation (ChIP) assays

ChIP assays were performed as described [43] using 3 µg of anti-Stra13 (Bethyl Laboratories) antibody. The following primers were used for amplification of the cyclin D1 promoter: 5′-GAGAGCTTAGGGCTCGTCTG-3′ and 5′-TGGGTGCGTTTCCGAGTAC-3′; and for β-actin promoter: 5′-GCTTCTTTGCAGCTCCTTCGTTG-3′ and 5′-TTTGCACATGCCGGAGCCGTTGT-3′.

#### Quantitative RT-PCR (Q-PCR)

Selected cells were synchronized in mitosis by adding nocodazole at a final concentration of 500 ng/ml. After 16 hr, nocodazole was removed by washing with media. Total RNA was extracted from cells using TRIZOL according to the manufacturer’s instructions (Invitrogen). Genomic DNA was eliminated by treatment with TURBO DNase (Ambion) and cDNA synthesis was carried out using AMV Reverse Transcriptase (Promega) according to manufacturer’s instructions. Q-PCR was performed using Light cycler 480 SYBR green I master (Roche) as described [43]. The following primers were used: Cyclin D1, 5′-AAGTGCGTGCAGAAGGAGATTGTG-3′ and 5′-TCGGGCCGGATAGAGTTGTCAGT-3′; p21, 5′-GCAGCCGAGAGGTGTGAGC-3′ and 5′-ACGGGACCGAAGAGACAACG-3′; cyclin B1, 5′-CGGTGAATGGACACCAACTCTG-3′ and 5′-CTGTGCCAGCGTGCTGATCT-3′; cyclin E1, 5′-TGTCCTCGCTGCTTCTGCTTTGTATCAT-3′ and 5′-GGCTTTCTTTGCTTGGGCTTTGTCC-3′; and GAPDH: 5′- ATCAACCGGGAAGCCCATCAC-3′ and 5′-CCTTTTGGCTCCACCCTTCA-3′.

### Luciferase Assays

Cells were transfected with the cyclin D1 promoter reporter pD1luc, Stra13, Stra13 2KR, SUMO1, SENP1 as indicated in the figures along with 5 ng of Renilla luciferase. Empty expression vector was added to normalize the amount of total DNA. 48 hr after transfection, luciferase activity was measured by the Dual-Luciferase Reporter Assay system (Promega). All transfections were performed in triplicates, and repeated at least twice.

### Immunofluorescence

COS-7 cells were used to examine subcellular localization of Stra13 and Stra13 2KR. 48 hr after transfection, cells were fixed in 4% formaldehyde, incubated with anti-Myc antibody and detected with secondary antibody coupled with Texas-red (Invitrogen). Slides were mounted in Vectashield (Vector Laboratories) supplemented with DAPI (4′, 6′-diamidino-2-phenylindole) to identify nuclei. Cells were visualized on a Zeiss LSM 510 META confocal laser-scanning microscope.

### Statistical Analysis

Error bars indicate mean ± standard deviation (S.D.). Statistical analysis was performed with by Student’s t-test and *P* values <0.05 were considered statistically significant [*p<0.05; **p<0.01].
